# Transient and Prolonged Response of Chicken Cecum Mucosa to Colonization with Different Gut Microbiota

**DOI:** 10.1371/journal.pone.0163932

**Published:** 2016-09-29

**Authors:** Jiri Volf, Ondrej Polansky, Karolina Varmuzova, Lenka Gerzova, Zuzana Sekelova, Marcela Faldynova, Vladimir Babak, Matej Medvecky, Adrian L. Smith, Bernd Kaspers, Philippe Velge, Ivan Rychlik

**Affiliations:** 1 Veterinary Research Institute, Hudcova 70, 621 00 Brno, Czech Republic; 2 Department of Zoology, University of Oxford, Tinbergen Building, South Parks Road, Oxford, United Kingdom; 3 Department for Veterinary Sciences, Institute for Animal Physiology, Faculty of Veterinary Medicine, Ludwig-Maximilians-Universität München, Veterinastr. 13, 80539 Munich, Germany; 4 INRA, Centre Val de Loire, UMR 1282 ISP Infectiologie et santé publique bat 311, 37380 Nouzilly, France; 5 Université de Tours, UMR1282 Infectiologie et Santé Publique, F-37000 Tours, France; University of Illinois at Urbana-Champaign, UNITED STATES

## Abstract

In this study we determined protein and gene expression in the caeca of newly hatched chickens inoculated with cecal contents sourced from hens of different ages. Over 250 proteins exhibited modified expression levels in response to microbiota inoculation. The most significant inductions were observed for ISG12-2, OASL, ES1, LYG2, DMBT1-L, CDD, ANGPTL6, B2M, CUZD1, IgM and Ig lambda chain. Of these, ISG12-2, ES1 and both immunoglobulins were expressed at lower levels in germ-free chickens compared to conventional chickens. In contrast, CELA2A, BRT-2, ALDH1A1, ADH1C, AKR1B1L, HEXB, ALDH2, ALDOB, CALB1 and TTR were expressed at lower levels following inoculation of microbiota. When chicks were given microbiota preparations from different age donors, the recipients mounted differential responses to the inoculation which also differed from the response profile in naturally colonised birds. For example, B2M, CUZD1 and CELA2A responded differently to the inoculation with microbiota of 4- or 40-week-old hens. The increased or decreased gene expression could be recorded 6 weeks after the inoculation of newly hatched chickens. To characterise the proteins that may directly interact with the microbiota we characterised chicken proteins that co-purified with the microbiota and identified a range of host proteins including CDD, ANGPTL6, DMBT1-L, MEP1A and Ig lambda. We propose that induction of ISG12-2 results in reduced apoptosis of host cells exposed to the colonizing commensal microbiota and that CDD, ANGPTL6, DMBT1-L, MEP1A and Ig lambda reduce contact of luminal microbiota with the gut epithelium thereby reducing the inflammatory response.

## Introduction

Vertebrates are hatched or born with a sterile intestinal tract and colonization is initiated as early as during hatch or delivery. The gut microbiota then develops further with the most dynamic changes in young animals and lower fluctuations in healthy adults. We recently characterized the life-time microbiota dynamics in egg laying hens [1] identifying an overall pattern of change that, except for a relative lack of *Actinobacteria* due to the absence of breast feeding in the chickens, resembles other animal species including humans [2,3].

The intestinal tract of any host responds to colonization with natural microbiota. For example, immunoglobulin production in the intestinal tract is dependent on the presence of microbiota since germ-free animals do not produce antibodies [4,5]. In chickens, low level changes in the amounts of mRNA encoding inflammatory cytokines have been reported between 2 and 5 days post hatch [6]. However, it is unlikely that these are the only host responses to microbiota colonization and the gut response to colonization by gut microbiota is far from being understood. The gut epithelia is covered with a double layer film consisting of mucin 2 (MUC2), IgA, Fc fragment of IgG binding protein (FcGBP), meprin 1A (MEP1A) and different antimicrobial peptides protecting epithelial cells from direct contact with gut microbiota [7]. However, the processes leading to the development of this protective layer during the initial phases of microbial colonization are not known.

Chickens represent a useful model for studies on the colonization of the intestinal tract since eggs and developing embryos are accessible for manipulation. In addition, chickens in commercial production are hatched from disinfected eggs in an extremely clean hatchery environment without contact with their parents. Inoculation of newly hatched chickens with gut microbiota of donor hens is a procedure with proven efficacy against colonization with pathogens [8]. Whilst this indicates the importance of healthy microbiota for the development of gut immune system, which genes, proteins and biological pathways are induced or suppressed following the colonization with natural microbiota chicken intestinal tract is not known.

In this study we therefore inoculated newly hatched chickens with cecal contents from donor hens of different ages and determined profiles of gene and protein expression in the cecum of naturally colonized hens and recipients of microbiota inoculation; a process that has not previously been studied in any system. This approach identified over 250 proteins with expression levels that altered in response to microbiota inoculation. Out of these, putative ISG12-2 protein (ISG12-2), immunoglobulins, fibrinogen-like domain (FReD) and cysteine rich scavenger domain (SRCR) containing proteins were the most prominent. Since FReD and SRCR proteins were secreted into gut lumen and found as tightly associated with gut microbiota, their induction is likely to be important for the interaction between microbiota and host within the mucin layer protecting intestinal epithelial cells from a direct contact with microbiota [7].

## Material and Methods

### Ethical statement

The animal work in the study was performed in accordance with current Czech legislation (Animal Protection and Welfare Act 246/1992). The specific experiments were approved by the Ethics Committee of the Veterinary Research Institute followed by the Committee for Animal Welfare of the Ministry of Agriculture of the Czech Republic (permit number MZe 1479). Animal experiments performed with germ-free and conventional chickens were carried out in strict accordance with French legislation and the specific protocol for the study on germ-free chickens was approved by the Val de Loire (N 2013/01/16) Ethics Committee for Animal Experiments.

### Sampling of chickens of different age and newly hatched chickens inoculated with microbiota of different composition

**Fig 1:**
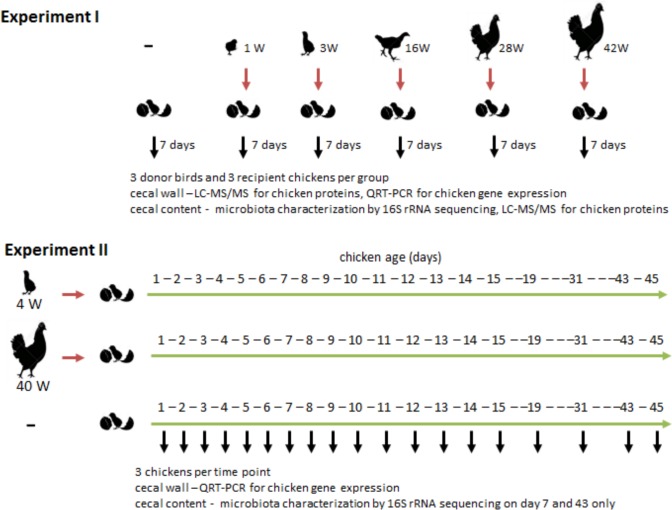
Experimental design and sampling strategy. Cecal contents of donor birds of different age were used for the inoculation of newly hatched (recipient) chickens. Cecal wall and cecal contents were always collected from both donor and recipient chickens and analyzed for microbiota composition, chicken protein and gene expression in the cecum, and secretion and association of chicken proteins with microbiota in gut lumen.

In the first experiment, ISA Brown chickens or hens 1, 3, 16, 28 and 42 weeks of age were obtained from a commercial farm, 3 birds of each age. Besides the characterization of microbiota composition and protein expression in their ceca, approx. 0.5 g of cecal content was collected from these birds and resuspended in 5 ml of PBS with 0.05% L-cysteine. After settling for 5 min the supernatant was decanted and a pool of equal volumes of the extracts from the donors of the same age was formed and 0.1 ml of this pool was orally applied to newly hatched chickens. Three chickens were inoculated on day of hatch with cecal microbiota extracts from donors of different ages and recipients were sacrificed 6 days later for sampling of cecal contents and cecal tissue (Fig 1). Chickens were reared in perforated plastic boxes with free access to water and the same feed and each experimental or control group was kept in a separate room. The whole experiment was accomplished on 3 different occasions. First we inoculated newly hatched chickens with cecal microbiota from 3- and 42-week-old donors, next we inoculated newly hatched chickens with cecal microbiota from 16- and 28-week-old donors and during the last sub-experiment we inoculated newly hatched chickens with cecal microbiota from 1-week-old donors.

### Inoculation of newly hatched chickens with microbiota from 4- or 40-week-old hens

In the second experiment, two groups of ISA Brown chickens, each consisting of 54 newly hatched chickens, were inoculated with cecal extracts of either 4- or 40-week-old donor hens (representing microbiota from young and old birds) on day of hatch. The third control group consisted of 54 non-inoculated ISA Brown chickens. In addition, 3 chickens were sacrificed on day 1 (the day of hatch) serving as a control to all 3 groups. On days 2, 3, 4, 5, 6, 7, 8, 9, 10, 11, 12, 13, 14, 15, 19, 31, 43 and 45 three chickens from each group were sacrificed and samples of cecal wall were taken into RNALater for qRT-PCR (see below). On days 7 and 43, cecal contents were also collected to check for the microbiota composition by sequencing of 16S rRNA genes as described below (Fig 1). Finally, 3 eggs at the last day of embryonal development, *i*.*e*. just a day before hatching, were obtained from a local commercial hatchery and cecal tissue of chicken embryos were collected into RNALater (these samples are designated as collected on day -1).

### Collection of samples from germ free chickens

Three naturally colonized and 3 germ free Leghorn chickens were sacrificed at 56 days of age and samples of cecal tissue were collected into RNALater for mRNA purification. Both groups originated from the same specific-pathogen-free (SPF) flock reared at the infectiology platform PFIE (INRA Val de Loire). The germ-free chickens were obtained by hatching and rearing chickens under sterile conditions as described [9] with some modifications. The surface of clean eggs, collected just after laying was sterilized by immersion in 1.5% Divosan for 5 min and for an additional 3 min just before eggs were transferred into a sterile HEPA-filtered incubator. After 18 days, the surface of eggs was sterilized in 1.25% Divosan for 4 min at 37°C. Eggs were then transferred in a sterile isolator for hatching, provided with a controlled ventilation and temperature. Temperature was maintained at 37.5°C for the first week and then reduced down by one degree per day to a stable temperature of 25°C. Birds were offered *ad libitum* X ray-irradiated starter diet from Special Diets Services (Dietex, Argenteuil France) and sterilized water for the entire duration of the experiment (56 days). The sterility of chickens was confirmed weekly by taking fresh fecal droppings which were incubated in tubes containing 10 ml of sterile brain-heart infusion broth under aerobic and anaerobic conditions. The conventional chickens were hatched from eggs collected the same day as the eggs for hatching germ-free chickens but incubated under conventional conditions. After 56 days, the conventional and germ-free chickens were sacrificed and cecal tissue was collected into RNALater for protein and RNA purification.

### Microbiota characterization

DNA was extracted from cecal contents using QIAamp DNA Stool Mini kit following manufacturer’s instructions (Qiagen). The purified DNA was stored at -20°C until further analysis. PCR was performed with forward primer 5’-*TCGTCGGCAGCGTCAGATGTGTATAAGAGACAG*-MID-GT-CCTACGGGNGGCWGCAG-3′ and reverse primer 5′- *GTCTCGTGGGCTCGGAGATGTGTATAAGAGACAG*-MID-GT-GACTACHVGGGTATCTAATCC-3′. The sequences in italics served for index and adapter ligation while underlined sequences represent sequences recommended for Illumina sequencing over V3/V4 region of bacterial 16S rRNA genes [10]. MIDs represent different sequences of 5–12 bp in length used for sample differentiation. PCR amplification and clean up were performed following manufacturer’s protocol using KAPA HotStart kit (Kapa Biosystems). Sequencing was performed using MiSeq Reagent Kit v3 chemistry according to the manufacturer’s instructions and the fastq files generated as an Illumina sequencing output were uploaded into Qiime software [11]. Quality trimming criteria were set to a value of 19, with no mismatch in the MID sequences and maximally 1 mismatch in the primer sequences. Chimera were identified and excluded by slayer algorithm [12]. The sequences were then classified with RDP Seqmatch with an operational taxonomic units (OTU) discrimination level set up to 97%. The raw sequence reads have been deposited in the NCBI Short Read Archive under the accession number SRP078556.

### Protein and RNA purification from chicken cecal tissue

Samples of chicken cecal tissue (50–100 mg) were used for parallel protein and RNA isolation. The samples were recovered from RNALater storage, mixed with 1 ml of TRI Reagent (MRC) and homogenized with MagNA Lyser (Roche). Fifty μl of bromoanisole was added to the homogenate and after centrifugation at 14,000 ×g for 15 min, proteins captured in the lower phenolic phase were precipitated with acetone. RNA present in upper aqueous phase (500 μl) was collected and mixed with an equal volume of 70% ethanol. This mixture was applied onto RNeasy purification columns and washing and RNA purification was performed exactly as recommended by the manufacturer (Qiagen). One μg of RNA was immediately reverse transcribed into cDNA using M-MLV reverse transcriptase (Invitrogen) and oligo (dT) primers.

### Detection of chicken proteins associated with cecal microbiota

To detect chicken proteins associated with cecal microbiota, the cecal contents (50–100 mg) of all donor hens and recipient chickens used in the first experiment were resuspended in 2 ml of 0.1% Tween 80, homogenized and centrifuged for 1 min at 50 g. Supernatant was transferred to a new tube and centrifuged at 4 000 g for 10 min. Washing of bacterial pellet with 0.1% Tween 80 was repeated 5 times. After the last washing step, the pellet was resuspended in 100 μl of 1% SDS and incubated at 100°C for 1 h. Subsequently, the protein lysate was mixed with 1.5 ml of TRI Reagent and processed as described above.

### Protein mass spectrometry

Protein pellets were dissolved in 300 μl of 8M urea and processed following the FASP protocol [13] using 10 kDa MWCO Vivacon 500 filtration device (Sartorius Stedim Biotech). Initial washing of proteins was performed with 8M urea followed by centrifugation for 12 min at 12,000 g. The reduction of the disulfide bonds was performed with 10mM dithiothreitol for 15 min at room temperature and acetylation was done with 50mM iodoacetamide for 15 min at room temperature. After 3 washings with 10mM triethylammonium bicarbonate, trypsin (Promega) was added at a 1:50 ratio and the digestion proceeded for 16 hours at 30°C.

LC-MS/MS analysis of tryptic peptides was performed using a Dionex UltiMate 3000 RSLC liquid chromatograph connected to a LTQ-Orbitrap Velos Pro hybrid mass spectrometer (Thermo Scientific). For each analysis, 5 μg of peptide sample was used. Each sample was separated on an EASY Spray C18 column (length 25 cm, I.D. 75 μm, particles 3 μm) using 300 nl/min flow rate of solvent A (0.1% formic acid) and solvent B (0.1% formic acid in 20/80 H_2_O/ACN (vol/vol)) and 150 min long reverse-phase gradient with concentration of solvent B increasing from 4% to 40%. From MS spectra (Orbitrap analyzer, 30,000 FWHM resolution, mass range 390–1700 m/z), the 10 most intensive peptides were fragmented using CID fragmentation (normalized collision energy 35) followed by MS/MS scan (LTQ analyzer). Raw LC-MS/MS data were analyzed using Proteome Discoverer v1.4. MS/MS spectra identification was performed by the SEQUEST algorithm using chicken protein sequence database. For each search, precursor and fragment mass tolerance were 10 ppm and 0.5 Da respectively. Only peptides with a false discovery rate (FDR) ≤ 5% were considered.

### Label free quantification

A list of proteins used in subsequent quantification was created by excluding those with low reproducibility in detection. For this purpose, peptide spectral matches (PSMs) for each protein were summed up using values for all the samples of the same group and proteins with total PSM lower than 5 were rejected. The proteins which were identified in only one chicken regardless of PSM counts were also excluded from subsequent analysis.

The proteins passing through the initial filtering criteria were arranged according to peak area in a descending order and peak areas were replaced by ranking, the most abundant being ranked as 1. Next, a geometric mean of each protein ranking in 3 chicken samples in each group was calculated. In the additional step we calculated interquartile range (IQR) for proteins ranked to the same position. Finally, to consider the protein as differentially regulated, difference in ranking of the particular protein in a certain chicken group must have exceeded 4 fold of theoretical IQR calculated for particular position and comparison of the groups using ANOVA followed by Tukey post-test must have showed statistical significance. In recipient chickens, the change in ranking was related to the ranking of particular protein in one-week-old donor chickens. Since no one group could be defined as biologically meaningful reference in donor chickens of different age, the change in ranking for particular protein was compared between the donor groups in which particular protein reached the highest and lowest ranking.

### Quantitative reverse transcribed PCR (qRT-PCR)

cDNA was diluted 10× with sterile water prior to the real-time PCR. qRT-PCR was performed in 3μl volumes in 384-well microplates using QuantiTect SYBR Green PCR Master Mix (Qiagen) and a Nanodrop pipetting station (Inovadyne) for PCR mix dispensing. PCR and signal detection were performed using a LightCycler II (Roche) with an initial denaturation at 95°C for 15 min followed by 40 cycles of 95°C for 20 s, 60°C for 30 s and 72°C for 30 s. Each sample was subjected to real time PCR in duplicate and the mean values of the duplicates were used for subsequent analysis. The Ct values of the genes of interest were normalized (ΔCt) to an average Ct value of 3 house-keeping genes, glyceraldehyde-3-phosphate dehydrogenase (GAPDH), ubiquitin (UB) and TATA box binding protein (TBP) and the relative expression of each gene of interest was calculated as 2^−ΔCt^. All the primers are listed in S1 Table.

### Data analysis and statistics

Principal Coordinate Analysis (PCoA) using proteomic and 16S rRNA sequencing data was calculated in R and in Qiime, respectively. ANOVA followed by Tukey post-test was used for the comparison of protein expression in the caeca of conventional and microbiota recipient chickens. In the real time PCR verifying the protein expression, only passing above threefold induction or suppression was considered as a threshold since the target genes were selected from significantly misregulated proteins. Gene expression in germ-free and conventional chickens was compared by the Mann-Whitney test. Differences in gene expression in time dependent experiment were calculated by Kruskal-Walis test followed by Dunn’s post-test using moving averages from 3 consequent time points from each comparison. In all cases, the calculations were performed with R software considering p < 0.05 as significance.

## Results

### Clustering of chickens according to microbiota composition and chicken protein expression in the cecum

**Fig 2:**
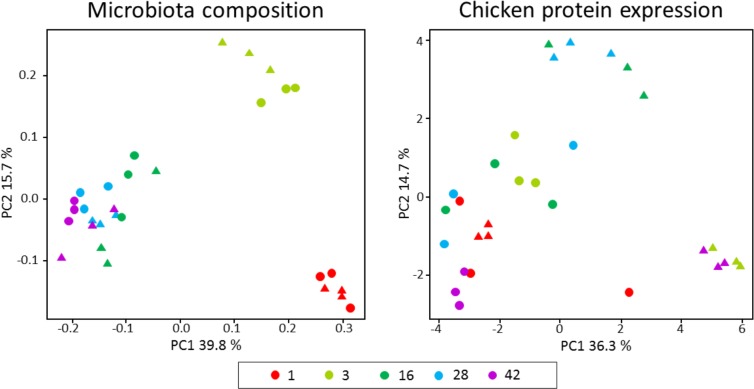
**Weighted Unifrac PCoA plot based on microbiota composition (A) or chicken protein expression (B) in each individual chicken or hen.** Circles, donor chickens and hens. Triangles, recipient chickens receiving microbiota of donors of particular age. Colors indicate chickens or hens of particular age in weeks or receiving microbiota from donors of particular age.

First we examined the interactions between the chicken and the cecal microbiota by monitoring microbiota composition and chicken cecal proteomic profiles in chickens that developed the microbiota under conventional conditions and in young chicks that were exposed to microbiota derived from different age donors. To address this question, we performed two PCoA analyses using either microbiota composition or chicken protein expression in individual chickens. Based on microbiota composition, chickens that developed microbiota under conventional conditions grouped according to age (Fig 2A). The recipients clustered tightly with the donor chickens of the respective age group (Fig 2A and (S2 Table). Interestingly, when proteomic profiles were compared in a similar analysis there were some changes in age-dependent profiles of conventional birds but the recipient chickens grouped into distinct clusters (Fig 2B). The donors and appropriate recipient chickens therefore expressed different proteins despite having microbiota of similar composition. Notably, the transplantation of microbiota from 1-week-old, 16- and 28-week-old, or 3- and 42-week-old chickens induced distinct proteomic profiles in the 7-day-old recipient chickens (Fig 2B).

### Identification of proteins differently expressed in conventionally colonized donors of different age or recipients inoculated with microbiota from donors of different age

The proteomic analyses identified 1,409 chicken proteins (S3 Table), of which 81 proteins were differentially expressed in conventionally colonized birds of different ages and 299 proteins were differentially expressed in the recipients following inoculation with microbiota from donors of different age (S4 Table). To test whether the proteomic signatures were a consequence of altered transcriptional activity, we targeted analyses on the 40 most up- or downregulated proteins identified in the donors of different age and 60 most up- or downregulated proteins in the recipients. Since some proteins represented highly regulated targets in both scenarios a total of 79 independent qRT-PCRs were applied to this analysis. Targets with more than 3 fold induction or suppression of mRNA levels in comparison to the expression levels in one-week-old conventionally colonized chickens included 37 of the 79 targets. Of these, 17 were expressed at higher levels than one-week-old chickens, 12 targets were at lower levels and 8 targets provided conflicting results depending on the experimental groups (Table 1). Expression of ISG12-2, 2'-5'-oligoadenylate synthetase-like (OASL), constant region of IgM immunoglobulin (IgM), immunoglobulin λ light chain (Igλ), ES1 protein homolog (ES1), cytidine deaminase (CDD), MRP-126 protein (MRP126), protein deleted in malignant brain tumor 1-like (DMBT1-L), angiopoetin related protein 6 (ANGPTL6), hemopexin (HPX), NK-lysin (NKL), ribonuclease homolog (RSFR) and lysozyme G-like 2 (LYG2) were induced by microbiota originating from at least 3-week-old chickens. Transcription of these genes increased also with age. Avidin (AVD), CUB and zona pellucida containing protein 1 (CUZD1) and aldose reductase (AKR1B10) were induced by microbiota originating from at least 3-week-old chickens but their expression did not change with age in conventionally colonized birds. Vitellogenin-2 (VTG2) was the only target where microbiota inoculation failed to induce changes in young chicks but was regulated according to age, consistent with its function in reproduction [14].

**Table 1:** List of genes with induced or suppressed transcript levels (>3-fold) in the chicken cecum inoculated with microbiota of different composition determined by qRT-PCR.

Gene name	MassSpec^#^	D1^$^	R1/D1^*^	R3/D1	R16/D1	R28/D1	R42/D1	D3/D1	D16/D1	D28/D1	D42/D1
ISG12-2	D&R	0.0269	**28.1**	**360.8**	**521.6**	**330.8**	**596.3**	**244.7**	**186.8**	**30.9**	**103.6**
OASL	R	0.0653	**6.7**	**22.9**	**45.6**	**39.8**	**66.7**	**29.5**	**15.4**	**4.9**	**4.2**
IgM	D	0.0259	2.8	**32.8**	**20.6**	**23.8**	**51.0**	**21.6**	**86.8**	**70.9**	**87.3**
Igλ	D&R	0.0001	1.2	**14.6**	**12.7**	**6.2**	**24.3**	**75.5**	**260.5**	**149.6**	**173.0**
ES1	R	0.0773	2.0	**16.2**	**15.0**	**11.0**	**30.3**	2.6	**3.1**	**4.4**	1.7
CDD	R	0.1809	2.0	**28.8**	**11.5**	**13.6**	**18.0**	**8.7**	**4.0**	**7.0**	1.7
MRP126	R	0.0010	**3.1**	**17.8**	**8.6**	**4.1**	**70.4**	**3.9**	1.5	**3.8**	**4.1**
DMBT1-L	R	0.0975	1.4	**23.1**	**8.5**	**11.0**	**15.0**	**5.5**	1.9	**5.7**	1.0
ANGPTL6	R	0.0003	-2.7	**12.1**	**8.0**	**6.2**	**8.7**	**8.2**	**3.9**	**9.2**	2.1
HPX	D	0.0214	-1.5	**6.8**	**3.2**	**4.3**	**4.1**	**3.8**	1.6	1.0	**10.5**
NKL	D	0.1797	2.2	**3.1**	**4.0**	**3.3**	**4.1**	**7.9**	**20.0**	**14.7**	**14.2**
RSFR	R	0.5421	1.2	**4.5**	2.0	1.4	**3.7**	**3.3**	2.2	2.4	**3.1**
LYG2	R	0.0062	2.1	**8.5**	**12.5**	**5.8**	**61.8**	**7.6**	1.7	**3.4**	1.7
AVD	R	0.2488	1.7	2.6	**17.3**	**10.6**	**45.4**	**-3.9**	2.3	1.1	2.0
CUZD1	D	0.0003	2.0	**4.8**	**4.3**	**3.7**	**5.6**	**4.3**	2.3	2.0	1.8
AKR1B10	R	0.0174	**8.3**	**8.0**	1.5	**5.7**	**6.2**	1.4	-1.5	2.2	-1.3
VTG2	D	0.0007	-1.1	1.6	2.1	1.8	-1.1	2.8	**4.5**	**3.2**	**208.7**
ALDOB	D&R	0.7466	-1.6	**-11.8**	**-4.0**	**-5.5**	**-7.8**	**-3.8**	-2.7	**-3.6**	-1.8
TTR	D	0.0175	-1.9	**-25.4**	**-49.1**	**-27.1**	**-33.1**	-2.9	-1.2	**-4.1**	2.3
CALB1	D&R	10.06	1.2	**-10.6**	1.8	**-5.1**	-2.5	-1.7	1.1	-1.3	**3.0**
ALDH1A1	D&R	8.68	-1.2	**-3.1**	**-3.9**	-2.2	**-4.4**	-2.7	-1.4	-2.0	1.1
ADH1C	R	12.66	1.1	**-3.7**	**-4.0**	**-3.5**	**-3.9**	-2.1	-1.4	-2.2	1.1
AKR1B1L	R	0.8796	-1.9	**-5.5**	**-7.6**	**-12.8**	**-10.6**	-2.5	-2.3	**-4.0**	-1.9
HSD17B2-L	R	4.0512	-1.3	-1.0	**-5.4**	**-3.1**	**-4.9**	-1.6	-2.8	**-3.1**	-2.1
BRT-2	D	55.3943	1.1	-2.6	-1.0	-1.8	-2.3	-2.1	**-3.0**	**-3.4**	**-3.5**
HEXB	D	1.89	1.1	-2.1	-1.1	1.1	-1.1	**-5.2**	2.1	**-3.5**	**-4.1**
CTSG	D	0.1682	2.3	1.3	1.6	1.0	-1.2	-1.1	-2.9	**-4.4**	**-4.2**
OIH	D&R	0.1263	-1.3	-1.3	-1.5	-2.1	-1.4	-1.9	**-4.3**	**-6.6**	**-4.7**
HBAD	D	0.1760	1.3	-2.1	-1.9	-1.5	**-6.3**	-2.8	**-19.0**	**-13.2**	**-12.1**
ATP5O^	R	1.0930	-1.1	1.1	1.4	1.0	-1.0	2.4	1.5	**-7.5**	1.1
ALDH2	R	9.77	-1.3	-3.0	-2.5	-2.3	**-3.5**	-1.7	1.0	-1.4	1.4
IFI30^	D	0.4229	-1.1	1.6	1.3	-1.0	1.5	2.2	**3.2**	2.8	2.5
CELA2A	D&R	0.0004	2.8	**3.8**	2.3	1.5	1.3	**3.4**	2.1	1.7	2.1
B2M	D&R	2.83	-1.1	-1.9	-1.0	-1.5	1.2	2.3	**3.4**	2.0	**5.1**
GC^	D	0.0030	-1.0	-2.9	-2.9	**-4.2**	**-9.1**	-2.6	-2.4	-2.5	**5.0**
LOC100857820	R	0.0224	1.2	2.0	-1.0	**3.1**	1.0	2.8	1.8	**4.4**	**3.9**
SERPINH1^	D	1.68	-1.0	-1.1	1.3	-1.1	-1.6	1.1	-1.9	**-3.2**	-2.2

# D or R indicates whether the particular protein was identified as differently expressed in donors of different age or recipients receiving microbiota from differently aged donors based on protein mass spectrometry

$ Data indicates reference expression in the caecum of 1-week-old donors detected by qRT-PCR

* Data show fold inductions of median values compared to the expression in 1-week-old donors, in red if induced and in blue is suppressed more than 3 fold

^ ATP5O, ATP synthase subunit O; GC, vitamin D-binding protein; IFI30, γ-interferon-inducible lysosomal thiol reductase; SERPINH1, serpin H1

Fructose-bisphosphate aldolase B (ALDOB) was the only gene with decreasing expression according to age and in recipients of microbiota from different ages while transthyretin (TTR), calbindin D28 (CALB1), retinal dehydrogenase 1 (ALDH1A1), alcohol dehydrogenase 1C (ADH1C), aldo-keto reductase family 1 member B1-like (AKR1B1L) and estradiol 17-β-dehydrogenase 2-like (HSD17B2-L) were down-regulated in recipients but not in birds of different ages. On the other hand, expression of β-tropomyosin (BRT-2), hexosaminidase B (HEXB), cathepsin G (CTSG), ovoinhibitor (OIH) and hemoglobin subunit α-D (HBAD) gradually decreased with increasing age and this effect was independent of microbiota inoculation (Table 1).

### Time-dependent expression in the cecum of recipients colonized by microbiota from 4- or 40-week-old donor hens

**Fig 3:**
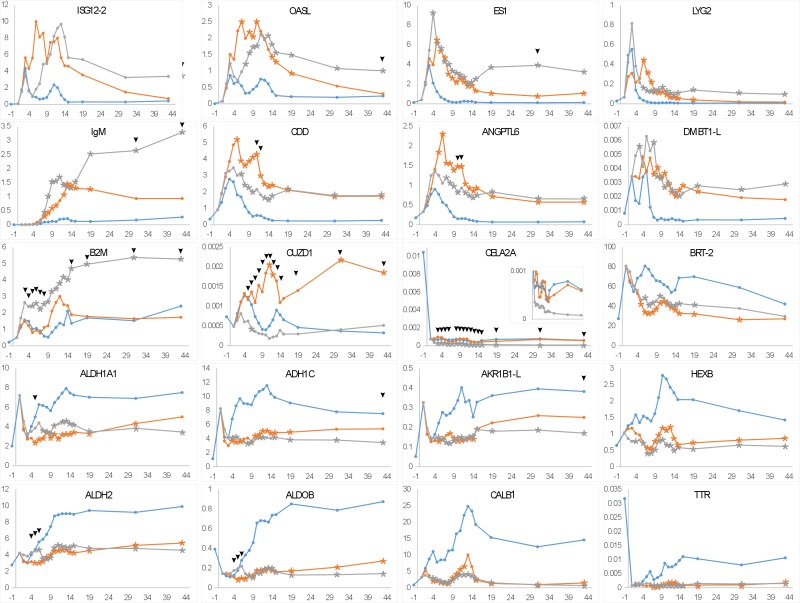
Expression dynamics of the genes responsive to the inoculation with cecal microbiota. Blue lines, expression in control, non-inoculated chickens. Red lines, expression in the chickens inoculated with microbiota from 4-week-old donor chickens. Grey lines, expression in the chickens inoculated with microbiota from 40-week-old donor chickens. Asterisks within the lines indicate significant difference from the expression in control, non-inoculated chickens. Arrowhead points to the significant differences in the expression in chickens inoculated with microbiota of 4- or 40-week-old donors. X-axis, age of chickens with -1 meaning the expression on the last day of embryonic development, i.e. a day before hatch. Y-axis, gene expression normalized to an average expression of 3 house-keeping genes. Data are displayed as moving average calculated from 3 consequent time points. Statistical significance was calculated by Kruskal-Walis test considering p<0.05 as significant.

Our results pertaining to the impact of inoculation of microbiota from different age donors resulted in identification of a range of age-independent microbiota-induced changes (Table 1) raises the interesting issue of how differences observed at 6 day post inoculation might impact on further development of the recipient chickens. In the next experiment we therefore verified and extended our earlier observations by characterization of gene expression in the cecum of chickens inoculated with microbiota of 4- and 40-week-old donors at regular intervals up to day 45 of life. The influence of inoculation with gut microbiota on cecal gene expression was confirmed for 36 genes and 20 the most significantly altered are shown in Fig 3.

In non-inoculated (conventionally colonized) chickens ISG12-2, OASL, ES1, LYG2 and, β2-microglobulin (B2M) exhibited peak expression in the cecum of 2-day-old chicks. DMBT1-L, CDD and ANGPTL6 reached their maximal expression at around day 3 to 6 of life and the first detectable expression of IgM and Igλ was recorded around day 10 (Fig 3). Expression of all these genes was induced following inoculation with gut microbiota. With ISG12-2, CDD and ANGPTL6, the response to the inoculation with microbiota of 4-week-old chickens was more pronounced during the first 10 days of life than following the inoculation with microbiota from 40-week-old hens. In contrast, the expression of OASL, ES1, B2M and IgM were significantly higher in chickens inoculated with microbiota of 40-week-old hens, while CUZD1 was induced only by microbiota from 4-week-old chickens, and except for B2M, this was detectable in recipient chickens older than 10 days (Fig 3).

The expression of mRNA encoding chymotrypsin-like elastase 2A (CELA2A), BRT-2, ALDH1A1, alcohol dehydrogenase 1C (ADH1C), AKR1B1L, HEXB, alcohol dehydrogenase 2 (ALDH2), ALDOB, CALB1 and TTR was suppressed following microbiota inoculation (Fig 3). HEXB, ALDH2, ALDOB, CALB1 and TTR were suppressed following the inoculation with either 4- or 40-week-old donor microbiota whereas CELA2A, ADH1C and AKR1B1L were suppressed only following the inoculation with microbiota from 40-week-old donors (Fig 3).

### Microbiota composition in inoculated and conventionally colonized chickens

**Fig 4:**
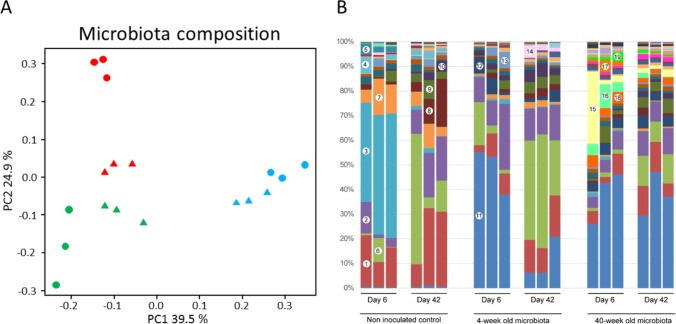
Microbiota composition in inoculated and conventionally colonized chickens. Panel A—Weighted PCoA plot based on microbiota composition in individual chickens at day 6 (small circles) and 43 (large circles) of life. The chickens inoculated with microbiota from 4-week-old donors are shown in green, from 40-week-old donors in blue and non-inoculated group is in red. Panel B–Composition of cecal microbiota of the individual chickens shown at genus level. 1 - [*Rumnimococcus*], 2—*Oscillospira*, 3—*Escherichia*, 4—*Akkermansia*, 5—*Proteus*, 6—*Faecalibacterium*, 7—*Blautia*, 8—*Lactobacilus*, 9—*Ruminococcus*, 10—*Coprococcus*, 11—*Bacteroides*, 12—*Parabacteroides*, 13—*Butyricoccus*, 14—*Streptococcus*, 15—*Prevotella*, 16—*Megamonas*, 17—*Mucispirillum*, 18—*Phascolarctobacterium*, 19—*Succinatimonas*.

To check for the efficiency of the inoculation and longer term stability of inoculated microbiota the microbial composition of cecal samples was determined in 7- and 43-day-old chickens that were conventionally colonized or were recipients of 4- or 40-week-old bird derived microbiota. The weighted PCoA (Fig 4A) analysis as well as microbiota composition at genus level (Fig 4B and S5 Table) confirmed the different composition in recipient and conventionally colonized groups. Microbiota of 6-day-old non-inoculated controls was dominated by *E*. *coli*. When chickens from this group reached age of 42 days, they were very similar to chickens inoculated with microbiota from 4-week-old donors. On the other hand, recipient chickens at 6 and 42 days post inoculation with microbiota from 40-week-old donors represented the most distinct groups (Fig 4).

### Expression of microbiota-inducible genes in germ-free chickens

Having established changes associated with administration of microbiota from different age chickens in recipients raised under conventional conditions we considered the impact of raising chickens in a germ-free environment. To identify which genes were microbiota dependent and which were regulated according to age in a microbiota-independent manner we analyzed gene expression in 56-day-old conventional and germ-free chickens. The qRT-PCR analyses revealed 6 genes with significantly lower expression in germ-free chickens compared to the conventionally colonized controls. These included AVD, IgM, Igλ, CALB1, ES1 and ISG12-2 (Table 2).

**Table 2:** Genes differently expressed in the caecum of germ-free and conventional chickens.

Gene name	Conventional	Germ free*	Conv/GF ratio	Mann-Whitney
AVD	0.060±0.057	0.00036±0.00103	166	p<0.01
Igλ	0.0096±0.0029	0.000086±0.00015	111	p<0.01
IgM	0.92±0.10	0.035±0.080	26	p<0.01
CALB1	5.78±0.84	1.87±0.468	3.1	p<0.01
ES1	0.19±0.28	0.073±0.0043	2.6	p<0.01
ISG12-2	1.06±0.42	0.49±0.22	2.2	p<0.05

* Data are shown as median±IQR

### Identification of bacteria-binding proteins

One of the levels of host-microbe interaction is the binding of host molecules to the surface to bacteria. Since previous studies with mammalian microbiota have identified DMBT1-L, LYG2 and immunoglobulins as proteins interacting with bacteria in extracellular space [4,15,16] next we employed a proteomics approach to identify which of microbiota inducible chicken proteins might be microbiota-associated proteins in a range of conventionally-colonized (or donor) chickens and in recipients of different treatments. After ranking the most abundant proteins adsorbed at the surface of gut microbiota, the ten most abundant from each group were selected and compared with their ranking in the whole cecal tissue (Table 3).

**Table 3:** Identification of chicken proteins associated with cecal microbiota.

	Rank in bacteria-bound proteins			Rank in cecal tissue			
	D1 & R1	D3-42	R3-42	D1 & R1	D3-42	R3-42	domains^#^
DMBT1-L	1*	7*	1*	640	453	129	FReD, SRCR
ANGPTL1-L	ND	4	2	ND	ND	ND	FReD
ANGPTL6	2*	2*	3*	653	435	127	FReD
DMBT1	7*	3*	4*	154	256	117	SRCR
CDD	14*	35*	5*	972	632	216	FReD
FGL1-L	ND	64	6	ND	ND	ND	FReD
MUC13	72*	13*	7*	1194	815	536	
AMY2A	4*	5*	8*	567	408	440	
LOC422270	60	8	9	ND	ND	ND	
ANPEP	17*	30*	10*	594	1006	587	
Igλ	ND	1*	18*	460	35	124	
MEP1A	24*	6*	29*	1014	964	1283	
PIGR	ND	9	186	ND	ND	ND	
COX2^	68*	10*	20*	242	386	506	
CPA1	3*	16*	11*	547	1167	1165	
SI	6*	26*	17*	1056	949	1066	
PRSS2	5*	14*	36*	182	940	918	
HBE1^	10*	66	48	77	93	133	
CPB1	9*	18*	63*	619	1256	1233	
SLC15A1^	8	184	145	ND	ND	ND	

* statistically significant difference between rank in bacteria-bound proteins and rank in caecal tissue (Mann–Whitney test, p < 0.05)

# Only SRCR and FReD domains are indicated

ND—Not detected

^ COX2, cytochrome c oxidase subunit 2; HBE1, hemoglobin subunit ε; SLC15A1, solute carrier family 15 member 1

Chicken proteins enriched the most by binding to the bacterial surfaces in recipient chickens receiving microbiota from at least 3-week-old donors included DMBT1-L, angiopoetin related protein 1-like (ANGPTL1-L), ANGPTL6, deleted in malignant brain tumor 1 (DMBT1), CDD, fibrinogen like protein 1-like (FGL1-L), mucin 13 (MUC13), amylase 2A (AMY2A), LOC422270 and aminopeptidase M (ANPEP). Six of them contained fibrinogen-like domain (FReD) or cysteine rich scavenger domain (SRCR). LOC422270 protein did not contain any known protein domain but exhibited partial similarity to β-subunit of meprin A of *Ophiophagus hannah*. When we checked for abundance of these proteins in the whole cecal tissue, these ranked to positions 100–600, or were not detectable. The same proteins were found also as commonly adsorbed to microbiota of donor chickens aged 3 weeks or more though in older chickens we detected Igλ as the most abundant chicken protein associated with cecal microbiota. In addition, polymeric immunoglobulin receptor (PIGR) and MEP1A were also among the 10 most abundant chicken proteins bound to cecal microbiota of 3-week-old or older chickens.

DMBT1-L, ANGPTL6, DMBT1, AMY2A and CDD were associated also with microbiota of 1-week-old chickens (chickens D1 and R1). However, due to the low expression of microbiota-inducible proteins in these chickens (Fig 3) or low affinity for binding to cecal microbiota, we detected additional proteins on the surface of the microbes from these birds including those most commonly associated with feed digestion including carboxypeptidase A (CPA1), carboxypeptidase B (CPB1), sucrase isomaltase (SI) or trypsin 2 (PRSS2).

## Discussion

In this study we determined response of chickens to inoculation with microbiota derived from different age donors (and of different composition). We identified 36 genes which were differentially expressed in the cecum at transcript and/or protein levels according to the nature of the inoculation. Of the genes suppressed by microbiota inoculation, ALDOB is a key enzyme in metabolism of carbohydrates. Its suppression may indicate a switch of gut epithelium from metabolism of glucose obtained from blood circulation as main energy source to microbiota-derived butyrate from gut lumen [17–19]. The suppression of TTR, transthyretin, a transport protein of thyroid hormones or retinol, is not contradictory to the situation in mammals, where TTR mRNA was identified in fetal intestinal tissue but not in adult rats [20,21]. The expression of ALDH1A1 (Retinal dehydrogenase 1) further supports the idea of modified retinal metabolism. Of the microbiota induced genes, IgM, Igλ, ES1, LYG2, MRP126, HPX, RSFR, AVD, OASL and NKL have also been reported as inducible following infection with a range of pathogens [16,22,23]. Their expression patterns suggest that the host may utilize similar pathways in responding to pathogens and in regulating the microbiota.

This study also identified microbiota-induced changes for a range of novel genes in cecal tissue including ISG12-2, CDD, DMBT1-L, ANGPTL6 or CUZD1. CUZD1 (CUB and zona pellucida-like domain-containing protein 1) was induced in the cecal tissue of chickens inoculated with microbiota of 4- but not 40-week-old donor hens. There is little functional information with CUZD1 except for the fact that anti-CUZD1 antibodies are used as a marker of inflammatory bowel disease in humans [24]. ISG12-2 was induced by microbiota inoculation and was also expressed at lower levels in germ-free compared with conventional chickens. Hence, the expression of ISG12-2 is directly dependent on microbial presence in the caecum. ISG12-2 localizes to mitochondria and its orthologue in mammals induces human cancer cells to become resistant to TRAIL-mediated apoptosis [25–27]. Interestingly, the renewal of senescent enterocytes in the crypt–villus axis is also controlled by TRAIL [28], therefore induction of ISG12-2 in the cecal tissue may protect enterocytes from TRAIL-mediated apoptosis following microbiota-mediated changes in the intestine.

The last group of microbiota induced proteins comprised CDD, DMBT1-L and ANGPTL6. All of these proteins contain FReD domain (DMBT1-L in combination with an SRCR domain) and such proteins were reported to be involved in aggregation. Mouse DMBT1, using its SRCR domain, directly binds and aggregates a variety of bacteria including *E*. *coli*, *Lactobacillus casei*, *Prevotella intermedia* and *Bacteroides fragilis* [29–31]. The FReD domains also bind and aggregate bacteria by saccharide binding [32,33]. In invertebrates, FReD containing proteins represent an evolutionary ancient defense mechanism whose main function is to agglutinate bacteria [34]. However, these proteins may also bind and aggregate food particles.

**Fig 5:**
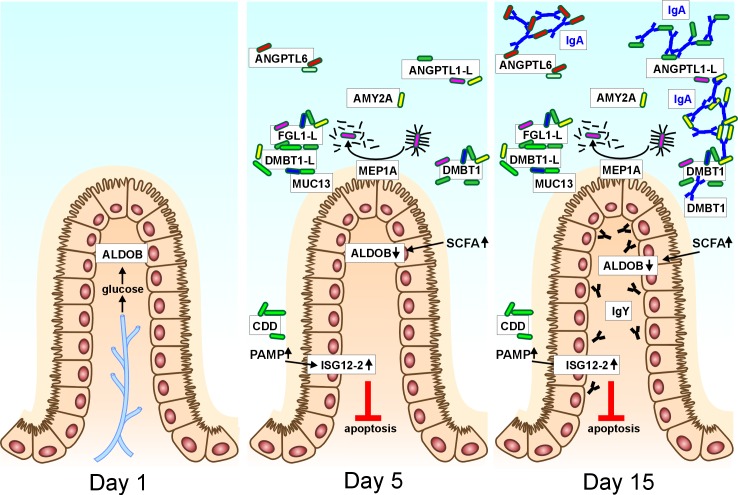
A model summarizing the interactions between the cecal microbiota and the chicken host during the early days of life. Chickens hatch with a sterile intestinal tract and the cecal epithelial cell metabolism is dependent on glucose from the circulation. Glycolytic enzymes like ALDOB are therefore highly expressed at Day 1 post hatch. Following cecal colonization, short chain fatty acids (SCFA) are produced by gut microbiota. This results in a switch from glucose to SCFA metabolism and decreased ALDOB expression. Cecal colonization also leads to epithelial cell exposure to bacterial components such as LPS or flagellin (both PRR-agonists). These induce upregulation of ISG12-2 which decreases the rate of epithelial cell apoptosis. Subsequently the chicken host responds to microbial colonization by increased expression of proteins with aggregative functions and meprin 1A protease (MEP1A) which cleaves bacterial fimbria. Bacterial aggregation or fimbria cleavage prevents extensive contact of commensal microbiota with chicken epithelial cells and uncontrolled inflammation (Day 5). From day 10 of life, immunoglobulins are produced in the chicken cecal tissue and IgA is translocated to the cecal lumen where it interacts with the microbiota (Day 15). Hence, our data can be summarized as a sequential set of interactions between the chicken ceca and the colonizing microbiota that affect both parties.

The identification of a range of upregulated proteins in the cecal tissue led us to consider the host proteome that was directly bound to the gut microbiota. MEP1A metalloproteinase, FReD or SRCR domain containing proteins and immunoglobulins were tightly associated with microbiota in gut lumen. MEP1A metalloproteinase is capable of cleavage of bacterial type I fimbria thus reducing bacterial adhesion to gut epithelium [35]. DMBT1 binds a number of chicken endogenous proteins such as secretory IgA, surfactant proteins A and D, complement factor C1q, lactoferrin, albumin or MUC5B [36–42]. The likely biological function of bacteria-adsorbed host proteins is to crosslink microbiota within the complex molecular network in the mucus and reduce their contact with enterocytes. With increasing age, the non-specific aggregative function of FReD and SRCR containing proteins is complemented by the secretion of IgA that may further shape the microbiota populations. Based on these observations we propose a hypothesis whereby cecal epithelial cells in the 1-day-old chicks that received no donor microbiota utilize glucose from blood circulation. Following microbiota colonization, enterocyte metabolism switches from utilization of glucose to butyrate or other short chain acids produced by gut microbiota. In addition, commensal bacteria induce ISG12-2 which protects host cells from extensive apoptosis. To reduce inappropriate contact between cecal microbiota and chicken epithelial cells, MEP1A and SRCR and FReD domain proteins are induced and secreted into gut lumen where these proteins adhere to the surfaces of gut microbiota. Microbial composition is finally shaped by secretion of immunoglobulins (Fig 5).

In conclusion we demonstrate that a single inoculation of microbiota on the day of hatch dramatically affects the cecal proteome of recipient chicks and that the nature of these changes is dependent upon the composition of the microbiome inoculation. The least dramatic changes were observed following inoculation with 1-week-old microbiota which are dominated by facultative anaerobes of phylum *Proteobacteria* whilst microbiota from older chickens are dominated by strict anaerobes [1]. These changes are long-lived and evident in recipients at 45 days of age, beyond the production period of broiler chickens. Early inoculation with microbiota represents an effective way of modulating the physiology of the host and reducing susceptibility to infection. Therefore these approaches have real potential to reduce the use of antibiotics in farmed animals, particularly in the poultry industry where production practices remove maternal microbiota interactions. Our work also has implications for other species where microbiome manipulation may result in substantial health benefits, not just in veterinary species but also in the human medicine.

## Supporting Information

S1 Table List of primers used for qRT-PCR in the study. (XLSX)

S2 Table List of OTUs identified in the first experiment using donor birds of different age and inoculated newly hatched chickens. (XLS)

S3 Table LC-MS/MS data exported from Proteome Discoverer supplemented with protein ranks in each experimental animal. (XLSX)

S4 Table ANOVA and Tukey post test analysis of mass spectrometry proteomic results. (XLSX)

S5 Table List of OTUs identified in the second time-dependent experiment with newly hatched chickens inoculated with microbiota from 4- and 40-week-old hens. (XLS)

## References

[1] Videnska P, Sedlar K, Lukac M, Faldynova M, Gerzova L, Cejkova D, et al Succession and replacement of bacterial populations in the caecum of egg laying hens over their whole life. PloS One 2014;9:e115142 10.1371/journal.pone.0115142 25501990 PMC4264878

[2] Mariat D, Firmesse O, Levenez F, Guimarăes V, Sokol H, Doré J, et al The Firmicutes/Bacteroidetes ratio of the human microbiota changes with age. BMC Microbiol 2009;9:123 10.1186/1471-2180-9-123 19508720 PMC2702274

[3] O’Toole PW, Claesson MJ. Gut microbiota: Changes throughout the lifespan from infancy to elderly. Int Dairy J 2010;20:281–291.

[4] Cebra JJ. Influences of microbiota on intestinal immune system development. Am J Clin Nutr 1999;69:1046S–1051S. 10232647 10.1093/ajcn/69.5.1046s

[5] Honjo K, Hagiwara T, Itoh K, Takahashi E, Hirota Y. Immunohistochemical analysis of tissue distribution of B and T cells in germfree and conventional chickens. J Vet Med Sci Jpn Soc Vet Sci 1993;55:1031–1034. 10.1292/jvms.55.1031 8117800

[6] Crhanova M, Hradecka H, Faldynova M, Matulova M, Havlickova H, Sisak F, et al Immune response of chicken gut to natural colonization by gut microflora and to Salmonella enterica serovar enteritidis infection. Infect Immun 2011;79:2755–2763. 10.1128/IAI.01375-10 21555397 PMC3191970

[7] Johansson MEV, Larsson JMH, Hansson GC. The two mucus layers of colon are organized by the MUC2 mucin, whereas the outer layer is a legislator of host-microbial interactions. Proc Natl Acad Sci U S A 2011;108 Suppl 1:4659–4665. 10.1073/pnas.1006451107 20615996 PMC3063600

[8] Methner U, Barrow PA, Martin G, Meyer H. Comparative study of the protective effect against Salmonella colonisation in newly hatched SPF chickens using live, attenuated Salmonella vaccine strains, wild-type Salmonella strains or a competitive exclusion product. Int J Food Microbiol 1997;35:223–230. 10.1016/s0168-1605(96)01236-6 9105931

[9] Schellenberg Maillard. Techniques d’élevage de volailles axéniques; in: Journées Rech Avicoles Cunicoles INRAITAVI- WPSA. Paris, ITAVI, 1973, pp 283–285.

[10] Polansky O, Sekelova Z, Faldynova M, Sebkova A, Sisak F, Rychlik I. Important metabolic pathways and biological processes expressed by chicken cecal microbiota. Appl Environ Microbiol 2015;82:1569–76. 10.1128/AEM.03473-15 26712550 PMC4771310

[11] Caporaso JG, Kuczynski J, Stombaugh J, Bittinger K, Bushman FD, Costello EK, et al QIIME allows analysis of high-throughput community sequencing data. Nat Methods 2010;7:335–336. 10.1038/nmeth.f.303 20383131 PMC3156573

[12] Haas BJ, Gevers D, Earl AM, Feldgarden M, Ward DV, Giannoukos G et al Chimeric 16S rRNA sequence formation and detection in Sanger and 454-pyrosequenced PCR amplicons. Genome Res 2011;21:494–504. 10.1101/gr.112730.110 21212162 PMC3044863

[13] Wiśniewski JR, Zougman A, Nagaraj N, Mann M. Universal sample preparation method for proteome analysis. Nat Methods 2009;6:359–362. 10.1038/nmeth.1322 19377485

[14] Li J, Leghari IH, He B, Zeng W, Mi Y, Zhang C. Estrogen stimulates expression of chicken hepatic vitellogenin II and very low-density apolipoprotein II through ER-α. Theriogenology 2014;82:517–524. 10.1016/j.theriogenology.2014.05.003 24938798

[15] Rosenstiel P, Sina C, End C, Renner M, Lyer S, Till A, et al Regulation of DMBT1 via NOD2 and TLR4 in intestinal epithelial cells modulates bacterial recognition and invasion. J Immunol 2007;178:8203–8211. 10.4049/jimmunol.178.12.8203 17548659

[16] Matulova M, Varmuzova K, Sisak F, Havlickova H, Babak V, Stejskal K, et al Chicken innate immune response to oral infection with Salmonella enterica serovar Enteritidis. Vet Res 2013;44:37 10.1186/1297-9716-44-37 23687968 PMC3663788

[17] Roediger WE. Role of anaerobic bacteria in the metabolic welfare of the colonic mucosa in man. Gut 1980;21:793–798. 10.1136/gut.21.9.793 7429343 PMC1419533

[18] Fitch MD, Fleming SE. Metabolism of short-chain fatty acids by rat colonic mucosa in vivo. Am J Physiol 1999;277:G31–40. 10409148 10.1152/ajpgi.1999.277.1.G31

[19] Clausen MR, Mortensen PB. Kinetic studies on colonocyte metabolism of short chain fatty acids and glucose in ulcerative colitis. Gut 1995;37:684–689. 10.1136/gut.37.5.684 8549946 PMC1382875

[20] Richardson SJ. Evolutionary changes to transthyretin: evolution of transthyretin biosynthesis. FEBS J 2009;276:5342–5356. 10.1111/j.1742-4658.2009.07244.x 19725882

[21] Yamauchi K, Ishihara A. Evolutionary changes to transthyretin: developmentally regulated and tissue-specific gene expression. FEBS J 2009;276:5357–5366. 10.1111/j.1742-4658.2009.07245.x 19725881

[22] Rychlik I, Elsheimer-Matulova M, Kyrova K. Gene expression in the chicken caecum in response to infections with non-typhoid Salmonella. Vet Res 2014;45:119 10.1186/s13567-014-0119-2 25475706 PMC4256799

[23] Barber MRW, Aldridge JR, Fleming-Canepa X, Wang Y-D, Webster RG, Magor KE. Identification of avian RIG-I responsive genes during influenza infection. Mol Immunol 2013;54:89–97. 10.1016/j.molimm.2012.10.038 23220072 PMC3565471

[24] Liaskos C, Rigopoulou EI, Orfanidou T, Bogdanos DP, Papandreou CN. CUZD1 and anti-CUZD1 antibodies as markers of cancer and inflammatory bowel diseases. Clin Dev Immunol 2013:968041 10.1155/2013/968041 23710207 PMC3654630

[25] Cheriyath V, Kuhns MA, Jacobs BS, Evangelista P, Elson P, Downs-Kelly E, et al G1P3, an interferon- and estrogen-induced survival protein contributes to hyperplasia, tamoxifen resistance and poor outcomes in breast cancer. Oncogene 2012;31:2222–2236. 10.1038/onc.2011.393 21996729

[26] Cheriyath V, Leaman DW, Borden EC. Emerging roles of FAM14 family members (G1P3/ISG 6–16 and ISG12/IFI27) in innate immunity and cancer. J Interferon Cytokine Res 2011;31:173–181. 10.1089/jir.2010.0105 20939681 PMC6468951

[27] Cheriyath V, Glaser KB, Waring JF, Baz R, Hussein MA, Borden EC. G1P3, an IFN-induced survival factor, antagonizes TRAIL-induced apoptosis in human myeloma cells. J Clin Invest 2007;117:3107–3117. 10.1172/JCI31122 17823654 PMC1964509

[28] Gassler N, Roth W, Funke B, Schneider A, Herzog F, Tischendorf JJW, et al Regulation of enterocyte apoptosis by acyl-CoA synthetase 5 splicing. Gastroenterology 2007;133:587–598. 10.1053/j.gastro.2007.06.005 17681178

[29] Bikker FJ, Ligtenberg AJM, Nazmi K, Veerman ECI, van’t Hof W, Bolscher JGM, et al Identification of the bacteria-binding peptide domain on salivary agglutinin (gp-340/DMBT1), a member of the scavenger receptor cysteine-rich superfamily. J Biol Chem 2002;277:32109–32115. 10.1074/jbc.M203788200 12050164

[30] Prakobphol A, Xu F, Hoang VM, Larsson T, Bergstrom J, Johansson I, et al Salivary agglutinin, which binds Streptococcus mutans and Helicobacter pylori, is the lung scavenger receptor cysteine-rich protein gp-340. J Biol Chem 2000;275:39860–39866. 10.1074/jbc.M006928200 11007786

[31] Madsen J, Tornøe I, Nielsen O, Lausen M, Krebs I, Mollenhauer J, et al CRP-ductin, the mouse homologue of gp-340/deleted in malignant brain tumors 1 (DMBT1), binds gram-positive and gram-negative bacteria and interacts with lung surfactant protein D. Eur J Immunol 2003;33:2327–2336. 10.1002/eji.200323972 12884308

[32] Wu C, Söderhäll K, Söderhäll I. Two novel ficolin-like proteins act as pattern recognition receptors for invading pathogens in the freshwater crayfish Pacifastacus leniusculus. Proteomics 2011;11:2249–2264. 10.1002/pmic.201000728 21598394

[33] Doolittle RF, McNamara K, Lin K. Correlating structure and function during the evolution of fibrinogen-related domains. Protein Sci 2012;21:1808–1823. 10.1002/pro.2177 23076991 PMC3575912

[34] Hanington PC, Zhang S-M. The primary role of fibrinogen-related proteins in invertebrates is defense, not coagulation. J Innate Immun 2011;3:17–27. 10.1159/000321882 21063081 PMC3031514

[35] Vazeille E, Bringer M-A, Gardarin A, Chambon C, Becker-Pauly C, Pender SLF, et al Role of meprins to protect ileal mucosa of Crohn’s disease patients from colonization by adherent-invasive E. coli. PloS One 2011;6:e21199 10.1371/journal.pone.0021199 21698174 PMC3116889

[36] Boackle RJ, Connor MH, Vesely J. High molecular weight non-immunoglobulin salivary agglutinins (NIA) bind C1Q globular heads and have the potential to activate the first complement component. Mol Immunol 1993;30:309–319. 10.1016/0161-5890(93)90059-k 8433709

[37] Ligtenberg AJM, Bikker FJ, De Blieck-Hogervorst JMA, Veerman ECI, Nieuw Amerongen AV. Binding of salivary agglutinin to IgA. Biochem J 2004;383:159–164. 10.1042/BJ20040265 15228387 PMC1134054

[38] Ligtenberg AJM, Karlsson NG, Veerman ECI. Deleted in Malignant Brain Tumors-1 Protein (DMBT1): A Pattern Recognition Receptor with Multiple Binding Sites. Int J Mol Sci 2010;11:5212–5233. 10.3390/ijms1112521 21614203 PMC3100851

[39] Tino MJ, Wright JR. Glycoprotein-340 binds surfactant protein-A (SP-A) and stimulates alveolar macrophage migration in an SP-A-independent manner. Am J Respir Cell Mol Biol 1999;20:759–768. 10.1165/ajrcmb.20.4.3439 10101009

[40] Oho T, Bikker FJ, Nieuw Amerongen AV, Groenink J. A peptide domain of bovine milk lactoferrin inhibits the interaction between streptococcal surface protein antigen and a salivary agglutinin peptide domain. Infect Immun 2004;72:6181–6184. 10.1128/IAI.72.10.6181-6184.2004 15385529 PMC517587

[41] Thornton DJ, Davies JR, Kirkham S, Gautrey A, Khan N, Richardson PS, et al Identification of a nonmucin glycoprotein (gp-340) from a purified respiratory mucin preparation: evidence for an association involving the MUC5B mucin. Glycobiology 2001;11:969–977. 10.1093/glycob/11.11.969 11744631

[42] Kojouharova MS, Tsacheva IG, Tchorbadjieva MI, Reid KBM, Kishore U. Localization of ligand-binding sites on human C1q globular head region using recombinant globular head fragments and single-chain antibodies. Biochim Biophys Acta 2003;1652:64–74. 10.1016/j.bbapap.2003.08.003 14580997

